# CRISPY-BRED and CRISPY-BRIP: efficient bacteriophage engineering

**DOI:** 10.1038/s41598-021-86112-6

**Published:** 2021-03-24

**Authors:** Katherine S. Wetzel, Carlos A. Guerrero-Bustamante, Rebekah M. Dedrick, Ching-Chung Ko, Krista G. Freeman, Haley G. Aull, Ashley M. Divens, Jeremy M. Rock, Kira M. Zack, Graham F. Hatfull

**Affiliations:** 1 Department of Biological Sciences, University of Pittsburgh, Pittsburgh, PA 15260 USA; 2 Department of Host-Pathogen Biology, The Rockefeller University, New York, NY 10065 USA; 3 Present Address: Department of Biomedical Sciences, West Virginia University, Morgantown, WV 26506 USA

**Keywords:** Prokaryote, Microbial genetics, Bacterial genetics, Mutagenesis, Bacteriophages

## Abstract

Genome engineering of bacteriophages provides opportunities for precise genetic dissection and for numerous phage applications including therapy. However, few methods are available for facile construction of unmarked precise deletions, insertions, gene replacements and point mutations in bacteriophages for most bacterial hosts. Here we describe CRISPY-BRED and CRISPY-BRIP, methods for efficient and precise engineering of phages in *Mycobacterium* species, with applicability to phages of a variety of other hosts. This recombineering approach uses phage-derived recombination proteins and *Streptococcus thermophilus* CRISPR-Cas9.

## Introduction

Bacteriophage genomics reveals massive genetic diversity and a vast abundance of functionally ill-defined genes^1^. Efficient and precise phage genome engineering is a critical step in understanding phage biology and in developing phages as effective therapeutics, diagnostics, and for numerous other applications^2–4^. We previously described Bacteriophage Recombineering of Electroporated DNA (BRED) as a method for engineering *Mycobacterium smegmatis* phages^5^, which has been subsequently adapted for phages of *Klebsiella*^6^, *Escherichia coli*^7^, and *Salmonella*^8^. In BRED, phage genomic DNA and a synthetic DNA substrate containing the desired mutation are co-electroporated into bacterial cells that express phage Che9c RecET-like recombination genes, *60* and *61*^9^, and plated for infectious centers on a bacterial lawn. Recombination is sufficiently efficient to enable identification of plaques containing mutant phage genomes by PCR without genetic selection, although these primary plaques typically contain both mutant and wild type phage particles and require further purification and screening^5^. Deletion of non-essential genes is often simple and relatively efficient using BRED; we previously showed that mixed primary plaques could be recovered from such deletions at an average frequency of 14% (range 4–60%)^5^. However, other types of recombinants such as larger deletions, replacements, and insertions are recovered at somewhat lower frequencies, demanding extensive screening of dozens or even hundreds of plaques. This is also observed when genome editing has deleterious impacts on phage growth^10^.

CRISPR-Cas systems provide defense against viral attack and are present in numerous bacterial and archaeal species^11^. Spacer sequences between repeat motifs in long arrays are transcribed to produce short RNAs (crRNAs) that together with Cas proteins target invading phage DNA for destruction through recognition of a protospacer corresponding to the crRNA. CRISPR-Cas systems are thus readily adapted for phage engineering, and have been used to modify phages that infect *Escherichia coli*^12^, *Streptococcus thermophilus*^13^ and *Vibrio cholerae*^14^ among others (Reviewed in Hatoum-Aslan, 2018)^15^. These previously described methods primarily rely on host-derived recombination functions and/or CRISPR-Cas^15^. The combination of highly efficient recombineering systems and CRISPR-Cas selection has been described for engineering of bacterial genomes^16^, and we describe a similar approach here for engineering phage genomes, taking advantage of the active and inactive Cas proteins described for genome editing and gene silencing (CRISPRi) in *Mycobacterium*^17,18^.

## Results

**Figure 1:**
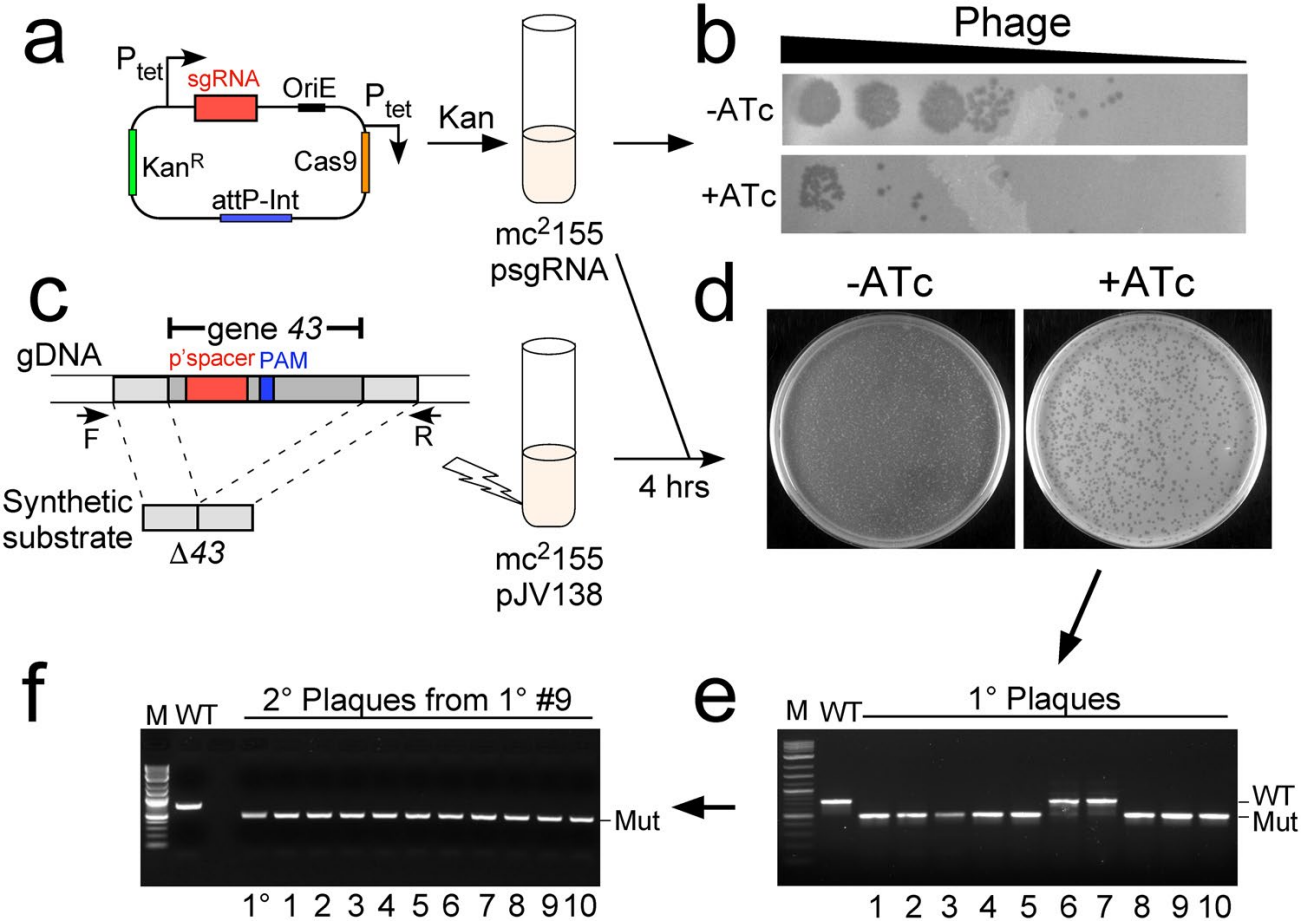
CRISPY-BRED phage engineering. (**a**). **I**n the first step of the CRISPY-BRED strategy, a CRISPR-Cas9 plasmid is constructed expressing a single guide RNA (sgRNA, red box) corresponding to a phage gene targeted for deletion or replacement (BuzzLyseyear gene *43* in this example); sgRNA expression is driven by a P_tet_ promoter and is inducible by addition of anhydrotetracycline (ATc). This plasmid is then introduced into *M. smegmatis* mc^2^155 selecting for kanamycin resistant transformants. (**b**) The sgRNA interference of phage infection is demonstrated by plating tenfold serial BuzzLyseyear dilutions on lawns of *M. smegmatis* transformants carrying the sgRNA plasmid (psgRNA) with (+ ATc) or without (-ATc) the inducer of sgRNA expression. In this example, the sgRNA reduces the efficiency of BuzzLyseyear plaquing approximately three orders of magnitude. (**c**) Two DNAs-BuzzLyseyear genomic DNA (gDNA) and a 500 bp synthetic DNA containing the sequences flanking gene *43*-are co-electroporated into *M. smegmatis* mc^2^155 carrying the recombineering plasmid pJV138 (mc^2^155pJV138). (**d)** After four hours recovery, the progeny are plated with *M. smegmatis* mc^2^155psgRNA cells in the presence of kanamycin (to counter select against the recombineering strain) to yield primary plaques in the presence or absence of ATc inducer. (**e**) Ten individual primary plaques from the + ATc plate were screened by PCR using forward and reverse primers (F, R, panel **c**), eight of which show a product corresponding to the desired mutant. (**f**) Phage particles from primary plaque #9 were diluted, replated on *M. smegmatis* mc^2^155 and ten secondary plaques screened by PCR, all of which have a mutant-sized product. CRISPY-BRIP differs in this overall strategy only in that the recombineering cells in panel **c** are electroporated with synthetic DNA substrate only, and the cell mixture is then infected with phage particles. Original gel images are shown in Supplementary Fig. 1.

We have combined BRED technology with CRISPR-Cas9 to facilitate efficient and precise phage genome engineering. In this approach, recombineering promotes recombinant formation and CRISPR-Cas9 targeting is used to counter-select against the parental (non-recombinant) phage (Fig. 1a). First, a plasmid derivative of pIRL53 (e.g. psgRNA) is constructed in which a single guide RNA (sgRNA) sequence is fused to an anhydrotetracycline (ATc)-inducible P_tet_ promoter. The plasmid contains a *Streptococcus thermophilus cas9* gene^17^, a kanamycin resistance gene, an *E. coli* replication origin, and an *attP*-*Int* cassette for chromosomal integration in mycobacteria^19^. The sgRNA component is typically 20 bp long and is designed to target either DNA strand within the parent phage only. The sgRNA sequence must be positioned 5′ to a Protospacer Adjacent Motif (PAM), and a variety of PAM sites with their relative activity in gene silencing has been described^17^. The efficiency of sgRNA targeting can be readily determined by reduction in plaque formation of the target phage on lawns of ATc-induced psgRNA-containing cells relative to uninduced cells (Fig. 1b). The reduction in plating efficiency varies from two to five orders of magnitude depending on the phage and the PAM/sgRNA (Fig. 1b, Table 1); the example shown in Fig. 1b indicates a reduction of 10^–3^ with induction of sgRNA expression. There is little or no difference in selection level if the transcribed or non-transcribed strand is targeted (Table 1).

**Table 1:** Recovery of engineered phages using CRISPY-BRED.

Phage^1^	Mut^2^	Gene Targets^3^	PAM^4^	sgRNA (strand)^5^	EOP^6^	1° plaq mut/tot^7^	2° plaq mut/tot^8^
Alma∆*ori*^9^	∆497 bp	*ori* ncRNA, *35*	NNAGAAA	sgRNA-1( +)	10^–2^	1/1	4/4
Alma∆*ori*^9^	∆497 bp	*ori* ncRNA, *35*	NNAGAAG	sgRNA-2(+ /−)^2^	10^–2^	ND	ND
BPs∆*32*-*33*_HRM^10^	∆1603 bp	*32* (*int*), *33* (*rep*)	NNAGAAT	sgRNA-3( +)	10^–3^	5/20	2/2
BPs∆*32*-*33*_HRM^10^	∆1603 bp	*32* (*int*), *33* (*rep*)	NNAGAAT	sgRNA-3( +)	10^–3^	18/20	ND
BuzzLyseyear∆*41*	∆117 bp	*41*	NNGGAAG	sgRNA-4( +)	10^–2^	10/10	10/10
BuzzLyseyear∆*42*	∆150 bp	*42*	NNGGAAC	sgRNA-5(-)	10^–2^	10/10	10/10
BuzzLyseyear∆*43*	∆240 bp	*43*	NNAGAAC	sgRNA-6( +)	10^–3^	8/10	10/10
BuzzLyseyear∆*43*	∆240 bp	*43*	NNAGGAT	sgRNA-7( +)	10^–3^	6/10	10/10
LadyBird∆*ori*^9^	∆400 bp	*ori* ncRNA, *34*	NNAGAAG	sgRNA-8(+ /−)^2^	10^–3^	ND	ND
LadyBird∆*ori*^9^	∆400 bp	*ori* ncRNA, *34*	NNAGCAT	sgRNA-9(+ /−)^2^	10^–4^	3/12	5/5
Miko∆r*epA*^9^	∆870 bp	*36* (*repA*)	NNAGAAA	sgRNA-10( +)	10^–2^	0/8	ND
Miko∆*repA*^9^	∆870 bp	*36* (*repA*)	NNAGAAG	sgRNA-11( +)	10^–2^	3/8	12/12
phiFW1^11^	∆279 bp	*14 (capsid)*	NNAGAAA	sgRNA-12( +)	10^–4^	1/12	4/4
Fionnbharth_ F52mut3	∆360 bp/ins. 273 bp	*47 (rep)/* F*52*mut3	NNGGAAA	sgRNA-13( +)	10^–3^	22/24	ND
Fionnbharth∆*45–47* mCherry	∆2509/ins. 1216 bp	*45 (int), 46, 47 (rep)/* mCherry	NNGGAAA	sgRNA-13( +)	10^–3^	16/16	ND
BPs∆*32*-*33_*HRM10 mCherry	∆1603/ins. 1216 bp	*32* (*int*), *33* (*rep*)/ mCherry	NNAGAAT	sgRNA-3( +)	10^–3^	14/18	ND
Adephagia∆*41–43* mCherry	∆2123/ins. 1216 bp	*41 (int), 42, 43 (rep)/* mCherry	NNAGAAG	sgRNA-14( +)	10^–3^	1/1	26/26

^1^Name of the desired phage recombinant.

^2^Mutation (Mut) indicates the type of mutation (∆; deletion, ins; insertion) and sizes thereof.

^3^Deleted/ inserted genes with putative gene functions in parentheses (int, integrase; rep, immunity repressor).

^4^The PAM site for each sgRNA is shown.

^5^For some constructions two sgRNA were tested for the same target genome; ( +) or (−) indicates whether the template ( +) or non-template (−) strand is targeted; (+ /−) indicates RNA transcripts are present on both strands. See Supplementary Table 1 for sequences.

^6^EOP, Efficiency of plaquing, as determined by differences in phage titers on ATc induced and uninduced media.

^7^Number of primary mutant plaques from the total tested. ND, Not Determined.

^8^Number of secondary mutant plaques from the total tested. ND, Not Determined.

^9^As described in reference 25^25^.

^10^Two slightly different BPs derivatives that carry host range mutations were engineered to have the same deletion.

^11^Phage phiFW1 is a derivative of phiTM45^26^, a derivative of Bxb1.

The synthetic dsDNA substrate used for BRED typically contains 150–250 bp of homologous sequence to the phage target on either side of the mutation, whether it is a deletion, replacement, or insertion (Fig. 1c). The dsDNA substrate and relevant phage genomic DNA (gDNA) are co-electroporated into recombineering-proficient cells that express recombination genes derived from phage Che9c (e.g. *M. smegmatis* mc^2^155pJV138) (Fig. 1d). Recovery is allowed for about 4 h, which is sufficient for completion of one round of viral lytic growth and the release of phage particles, which have either wild type or mutant genomes. This is a departure from the BRED method where recovery is shorter than a lytic cycle of growth. The mixture of recombineering cells and phage is then plated together with the selective *M. smegmatis* mc^2^155psgRNA strain onto solid media containing kanamycin to prevent growth of the recombineering strain (Fig. 1d). Plating this mixture onto solid media lacking ATc permits replication and plaquing by both wild type and mutant phage derivatives, but plating onto solid media containing ATc induces expression of the CRISPR-Cas system and selects against replication of wild type phage genomes. In the absence of ATc plaques derived from both wild type and mutant particles are efficiently recovered (and can give near-confluence lysis as in Fig. 1c), but sgRNA-expression reduces wild type growth, enriching for desired recombinant phage and any other variants escaping CRISPR-Cas selection. Thus, plating in the presence and absence of ATc indicates the strength of counter-selection against the parent phage. The number of plaques recovered on the ATc plate depends on the efficiency of both electroporation and recombination, but is usually from dozens up to several hundred. Individual plaques are picked from the ATc plate and screened by PCR to detect the mutant allele (Fig. 1e). The proportion of mutant plaques varies somewhat from 20–100% but typically requires screening of not more than a dozen plaques, which by PCR appear to be homogenous (Fig. 1e, Table 1). This is another departure from BRED, where these primary (1°) plaques are always mixtures of wild type and mutant alleles, requiring at least one more round of purification and screening. In contrast, re-plating and re-screening of secondary (2°) plaques generated by CRISPY-BRED confirms that all are mutant and that the primary plaques are homogenous (Fig. 1f, Table 1). Table 1 describes the generation of thirteen phages with deletions or insertions by CRISPY-BRED. Genome sequencing of four of these (Alma∆*ori*, LadyBird∆*ori*, Miko∆*ori*, and BuzzLyseyear∆*43*)*,* and several other CRISPY-BRED recombinants have revealed no off-target mutations, suggesting that untargeted additional mutations are uncommon.

As shown in Fig. 1e, a small proportion of primary plaques can give the PCR product expected from the parent phage. These plaques reflect one of two outcomes: phenotypic escape of the parental genome from CRISPR-Cas selection, or non-recombineering-directed mutants (such as point mutations or small insertions/deletions) that reduce or eliminate CRISPR-selection but give parental-sized PCR products. Such mutations could pre-exist in the population, or result from repair events after post-CRISPR-Cas cleavage^20^.

The primary advantage of CRISPY-BRED over BRED is that it simplifies recovery of recombinants when recombination is less efficient. For the phages described in Table 1, recombination efficiencies were calculated for five of them. For BuzzLyseyear∆*41* and BuzzLyseyear∆*42*, recombination occurred readily, with efficiencies of 2 × 10^–1^ and 3 × 10^–1^; to isolate these mutants, typical BRED would likely have been sufficient. However, phages Alma*∆ori*, LadyBird*∆ori,* and Miko*∆repA* were generated at efficiencies of 6 × 10^–2^, 8 × 10^–3^ and 2 × 10^–3^, respectively, such that CRISPR-Cas selection against parental phage permitted identification of the desired mutants in screening of a handful of plaques, rather than tens or hundreds otherwise.

**Figure 2:**
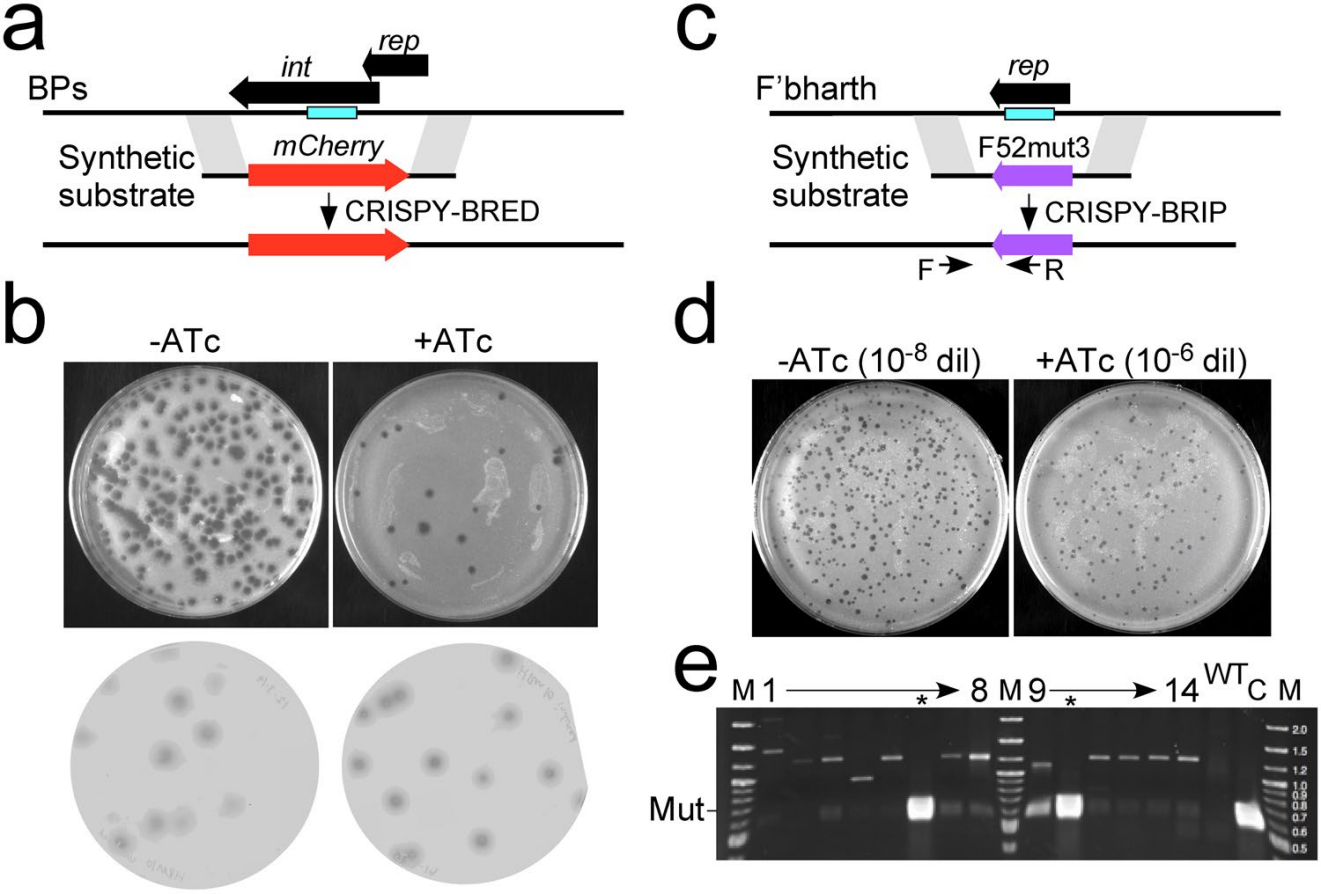
Construction of engineered phages. (**a**) Scheme for constructing an mCherry reporter derivative of phage BPs. A synthetic substrate containing the mCherry gene and sequences flanking the BPs integrase-repressor (*int*-*rep*) was designed to replace the *int*-*rep* region. The blue box indicates the position of the protospacer and PAM site to which a sgRNA was designed. (**b**) Primary plaques (top) were recovered with (+ ATc) or without (-ATc) inducer following co-electroporation of the synthetic DNA substrate and phage BPs genomic DNA into *M. smegmatis* recombineering cells and plated with *M. smegmatis* psgRNA cells (see Fig. 1). Fluorescent images of the plaques (bottom) show similar numbers of mCherry recombinants (dark plaques) in the presence (+ ATc) or absence (−ATc) of inducer, but these represent > 90% of all plaques recovered when the sgRNA is expressed (+ ATc). (**c**) CRISPY-BRIP engineering, illustrated by construction of a recombinant Fionnbharth phage carrying Fruitloop *52*mut3 (F*52*mut3). A synthetic substrate containing Fruitloop *52*mut3 (F*52*mut3) and sequences flanking the repressor (*rep*) gene of Fionnbharth (F’bharth) was constructed such as to replace the Fionnbharth repressor gene. The blue box indicates the position of the protospacer and PAM site to which a sgRNA was designed. (**d**) Primary plaques were recovered with (+ ATc) or without (-ATc) inducer following electroporation of the synthetic DNA substrate into *M. smegmatis* recombineering cells, followed by infection of Fionnbharth particles, incubation to allow a round of lytic growth, and plating with *M. smegmatis* psgRNA cells (see Fig. 1); samples were diluted 10^8^-fold or 10^6^-fold and plated with or without ATc, respectively. (**e**) PCR of 14 plaques from the + ATc plate using forward and reverse primers (F and R, panel **c**) primers identified two recombinant plaques (asterisks); wild type phage (wt) and control (C) DNAs are shown. Original gel images are shown in Supplementary Fig. 1.

An additional example of CRISPY-BRED utility is the construction of fluorescent reporter phages, such as replacement of the immunity cassette (*int-rep*) of phage BPs with mCherry (Fig. 2a). Recombineering alone yields desired recombinant progeny at < 3% of the primary plaques, which would require extensive plaque screening (Fig. 2a, b). In contrast, > 90% of the plaques recovered by CRISPY-BRED are recombinant (Fig. 2b). Although in this instance fluorescence indicates the desired recombinants, this broadly reflects the benefit of CRISPY-BRED in constructions where recombinants are formed at low efficiency and a visual marker is not available. An example is provided by phiFW1, a capsid variant of phage Bxb1, which is viable but still rare among CRISPY-BRED progeny (Table 1). This is facilitated by strong counter-selection, and this derivative could not have been readily constructed using BRED alone (Table 1). We note that in this example, sequencing of non-recombineering directed survivors showed they are CRISPR-escape mutants with protospacer mutations or deletions (data not shown).

The combination of CRISPR-mediated counter-selection and recombineering also provides an opportunity for engineering phages that transfect inefficiently. Instead, phage genomes are provided by infection, as has been described previously to recombineer *E. coli* phages^21^. To evaluate this adaptation (BRIP: Bacteriophage Recombineering with Infectious Particles), we electroporated a synthetic DNA substrate into recombineering cells, infected these with phage particles, and incubated for 4.5 h to allow a cycle of lytic growth (Fig. 2c). The parental phage was Fionnbharth and the DNA substrate was designed to replace gene *47* (*rep*) with a gene *52* variant from Fruitloop (F52mut3)^22^. Plaque recovery was reduced by 100-fold when plated on solid medium with ATc (Fig. 2d), and PCR screening of 14 plaques identified two desired recombinants (Fig. 2e). Using the same configuration with CRISPY-BRED (co-electroporating gDNA and mutant substrate), 22 of 24 plaques screened were recombinant (Table 1). CRISPY-BRIP is less efficient than CRISPY-BRED, as expected, but useful when gDNA electroporation is inefficient. The CRISPR-mediated counter-selection greatly enhances the ease of recombinant identification.

CRISPY-BRED and CRISPY-BRIP have been used to construct many phage recombinants using different phages and different types of mutations (Table 1). Not all mutants are viable (data not shown), but viability is simpler to determine with CRISPY-BRED relative to BRED alone, because if CRISPR selection is strong, then all of the plaques recovered are either CRISPR escape mutants or the desired recombinants. For example, an attempt to modify the C-terminus of the tail tube protein (gp19) of Bxb1 resulted in 16/16 plaques with protospacer deletions but none of the desired mutant, suggesting it is non-viable (data not shown). The strength of CRISPR-mediated counter-selection is somewhat variable, and for some phages is minimal (< 10%); expression of anti-CRISPR proteins could account for this, but have yet to be identified in mycobacteriophages. A variety of PAM and sgRNA sequences could be evaluated to optimize counter-selection for a particular phage if needed.

## Discussion

A potential limitation of CRISPY-BRED are the constraints imposed by PAM site choice. There is a 5 bp requirement for this Cas9 system and although many variants can be used^17^ these may not be present at precise locations where it is desired. For deletions and replacements where there is a reasonably long DNA region (> 300 bp) for targeting, this should not be problematic, but for point mutations and precise insertions (rather than replacements), PAM site choice could be limiting. Alternative CRISPR-Cas systems with more lax PAM requirements^23^ could be adapted for more facile CRISPY-BRED applications. A second potential disadvantage of CRISPY-BRED relative to BRED is the necessity to design, construct and transform the sgRNA plasmid. In practice, this does not take substantially longer than the design, synthesis, and amplification of the dsDNA substrate, and has the substantial advantage of simplifying recombinant identification.

We note that the magnitude of phage interference by CRISPR-Cas varies substantially depending on the phage and the sgRNA used (Table 1). The reason for this is not clear but presumably reflects at least in part the efficiency of PAM site recognition^17^; however, it could also potentially be influenced by phage-encoded anti-CRISPR genes^24^. It seems unlikely that plaques escaping selection have altered or mutant protospacer or PAM sequences, when plaquing is only reduced 100-fold. However, even a modest (100-fold) reduction in plaquing with CRISPR-selection greatly increases the ease with which desired recombinants can be identified.

CRISPY-BRED and CRISPY-BRIP should be applicable to bacteriophages for a variety of other bacterial hosts. CRISPR-Cas systems are readily adaptable^11^ and counter-selection can be relatively poor (two orders of magnitude reduction) and still be effective for mutant enrichment. Recombineering systems may be more limiting for other bacteria, but many phages code for their own lambda Red-like or RecET-like recombination systems, and can be co-opted for recombineering system development as for the mycobacteria^9^. Some phages-especially those with relatively large genomes-do not efficiently transfect their bacterial hosts, and the CRISPY-BRIP adaptation offers a useful approach to precisely and efficiently engineer these phages.

## Methods

Detailed methods for CRISPY-BRED and CRISPY-BRIP strategies are described in the online Methods section.

## Supplementary Information

Supplementary Information

## Data Availability

The genome sequences of mycobacteriophages referenced here are available at phagesdb.org.
