# Supplementary material for: CRISPY-BRED and CRISPY-BRIP: efficient bacteriophage engineering
Source: Sci Rep. 2021 Mar 24;11:6796. doi: 10.1038/s41598-021-86112-6 (PMC7990910; doi:10.1038/s41598-021-86112-6)
Supplement: Supplementary file 1 — Supplementary Information [file 41598_2021_86112_MOESM1_ESM.pdf]

SUPPLEMENTARY MATERIALS

CRISPY-BRED and CRISPY-BRIP: Efficient bacteriophage engineering

Running Title: CRISPY-BRED

Katherine S. Wetzel†, Carlos A. Guerrero-Bustamante†, Rebekah M. Dedrick†, Ching-Chung Ko, Krista G. Freeman, Haley G. Aull, Ashley M. Divens[3], Jeremy M. Rock[2], Kira M. Zack, and Graham F. Hatfull*

[1]Department of Biological Sciences
University of Pittsburgh
Pittsburgh, PA 15260

Department of Host-Pathogen Biology
The Rockefeller University
New York, NY 10065

[3]Current address:
Department of Biomedical Sciences
West Virginia University
Morgantown, West Virginia 26506, USA

*Corresponding author; gfh@pitt.edu

†These authors contributed equally

Online Supplementary Methods

Bacterial strains and media

All experiments described here were done in *M. smegmatis* mc^{2}155[18]. *M. smegmatis* mc^{2}155 strains were grown in Middlebrook 7H9/10% Albumin Dextrose Complex (ADC)/ 50 µg/mL carbenicillin (CB)/10 µg/mL cyclohexamide (CHX)/1 mM $CaCl_2$ unless otherwise noted. Tween-80 (0.05%) was used in starter cultures but omitted in subcultures used for phage infections.

Design of CRISPR-Cas9 selection strains

Appropriate PAM and protospacer sequences in the region to delete were identified using the parameters described in Rock *et al*[12]. Briefly, the region to be deleted was screened for the presence of one of the 15 PAM sequences with >25-fold knockdown of a *Renilla* target by the *S. thermophilus* dCas9. Oligonucleotides corresponding to the sgRNA targeting sequence adjacent to the chosen PAM were annealed and ligated using T4 ligase (NEB) into pIRL53 (Kan^R) that had been digested with BsmBI. The sgRNA targeting sequences in these psgRNA plasmids are shown in Supplementary Table 1. The ligated plasmid was used to transform DH5a *E. coli* cells and miniprepped. The resulting plasmid (30-100ng) was transformed into 100 µL electrocompetent *M. smegmatis* mc^{2}155 using the following parameters: 2.5 kV, 25 uF, 1000 ohms. Electroporated cells were recovered by shaking for 2-4 hours at 37° C in 7H9/ADC and then plated on Middlebrook 7H10/0.2% dextrose/0.5% glycerol/1 mM $CaCl_2$/20 µg/mL kanamycin (Kan) solid media plates. The resulting colonies were first grown as starter cultures in 7H9/ADC/Tween/ 20 µg/mL Kan/ $CaCl_2$, and then subcultured for use in CRISPY-BRED in 7H9/ADC/Kan/$CaCl_2$.

Design of recombineering substrate

Recombineering substrates were generated as previously described[5]. Briefly, to delete a genome segment, the substrate consisted of a synthesized dsDNA gBlock (IDT) containing approximately 250bp upstream and 250bp downstream of the segment to be deleted.

To replace a genome segment with an mCherry expression cassette, two dsDNA gBlocks were synthesized that consisted of either ~250 bases upstream or ~250 bases downstream of the insertion site; each gBlock also contained ~20 bases of homology to an mCherry cassette to permit Gibson assembly. The mCherry cassette used here was originally described as an insert in plasmid pLO87[19,20]. To generate the recombineering substrate, the two gBlocks and the mCherry insert were assembled at a molar ratio of 1:1:2 using the NEBuilder HiFi DNA Assembly kit (NEB). The assembly product was then amplified using primers that annealed to the 5' and 3' ends of the product to generate sufficient quantities for recombineering. Other gene replacements were made similarly.

CRISPY-BRED

Induced electrocompetent *M. smegmatis* $mc^2$155pJV138 recombineering cells were prepared similarly to *M. smegmatis* $mc^2$155pJV53 cells described previously[5]. Plasmid pJV138 is similar to pJV53, except it encodes a hygromycin (Hyg) resistance marker rather than a kanamycin (Kan) resistance marker. Briefly, 100 mL of *M. smegmatis* $mc^2$155pJV138 was grown in 7H9/CHX/Hyg (150 µg/mL)/Tween/ 0.2% succinate to an OD_{600} of 0.4-0.5. Then, to induce Che9c *60* and *61* expression, acetamide was added to 0.2% and cells incubated shaking at 37° C for 3 additional hours. Cells were then incubated on ice for 30-120 mins, washed three times with sterile, ice-cold, 10% glycerol, resuspended in ~5% original volume in 10% glycerol, aliquoted and flash frozen for storage at -80° C.

100μL of recombineering cells were thawed and transformed via electroporation with 50-200 ng phage genomic DNA and 100-400 ng BRED substrate. Cells were recovered in 1 mL 7H9/ADC/$CaCl_2$ at 37° C for at least 4 hours to permit lysis and release of mature phage particles. The recovered cells were then combined with the *M. smegmatis* $mc^2$155psgRNA (i.e. *M. smegmatis* $mc^2$155 carrying a derivative of plasmid pIRL53) selection strain and plated with top agar onto 7H10/ADC/Kan/$CaCl_2$ or 7H10/glycerol/dextrose/Kan/$CaCl_2$ solid media with and without anhydrotetracycline (ATc, 100 ng/mL). Either one transformation was performed and split onto two solid media plates (+/- ATc), or two parallel transformations were performed where one was plated onto solid media with ATc, and one without. Plates were incubated at 37° C for 24-48 hours.

Well-isolated plaques were picked from the +ATc plate into 100 μL phage buffer (10 mM TrisHCl, pH 7.5; 10 mM $MgSO_4$; 68.5 mM NaCl; 1 mM $CaCl_2$). 1-2 μL were used to screen the plaque by PCR (NEB Q5 2X Master Mix) for an amplicon of the appropriate size using primers that flank the intended deletion or replacement. Plaques that yielded a band of the expected size were plaque purified for one additional round, and then plated onto solid media to generate a plate with confluent lysis from which phage were recovered for use as a high titer lysate.

CRISPY-BRIP

CRISPY-BRIP was performed the same way as CRISPY BRED, with the following modifications: At the electroporation step, only BRED substrate was electroporated into recombineering cells. Cells were then recovered in 1mL 7H9/ADC/$CaCl_2$, and 10uL of Fionnbharth lysate was added (approximately 10, 100 or 1000 PFU). Phage adsorbed at room temperature for 10 minutes, then cells were incubated at 37°C shaking for 4.5 hours. Cells were then plated with the CRISPR-Cas selection strain as described for CRISPY-BRED.

Supplementary Table

Supplementary Table 1. sgRNA targeting sequences used in CRISPY-BRED

sgRNA[1]	Parent Phage[2]	PAM[3]	sgRNA targeting sequence (strand)[4]
sgRNA-1	Alma	NNAGAAA	GTGATACGAGTGACCTTGGC (+)
sgRNA-2	Alma	NNAGAAG	GGCCCATCCACCTCGGATAG (+/-)
sgRNA-3	BPs and derivatives	NNAGAAT	CGAGCGAGTCGAGATAGTCGT (+)
sgRNA-4	BuzzLyseyear	NNGGAAG	TGGGCATGTGTGGTGCACTG (+)
sgRNA-5	BuzzLyseyear	NNGGAAC	TTGACGCAGCCCACTGGCAG (-)
sgRNA-6	BuzzLyseyear	NNAGAAC	CTCGGATGTGTTCGGGTCCA (+)
sgRNA-7	BuzzLyseyear	NNAGGAT	CTCTCCCGCGTGAATACCCC (+)
sgRNA-8	LadyBird	NNAGAAG	GGCCCATCCACTGGGGATAG (+/-)
sgRNA-9	LadyBird	NNAGCAT	TGGCAGCGTAGCGGTGGCCG (+/-)
sgRNA-10	Miko	NNAGAAA	CCCGCCTTCGGCGGTAGGTA (+)
sgRNA-11	Miko	NNAGAAG	GATCCCTAACCCAGAGAGCC (+)
sgRNA-12	Bxb1 and derivatives	NNAGAAA	GTACGTGTCAAGTTGTCAGCCG (+)
sgRNA-13	Fionnbharth	NNGGAAA	CGTACTCGATTGCGGATTGC (+)
sgRNA-14	Adephagia	NNAGAAG	TTCCTTCACCTCCCGCTCCC (+)

[1] sgRNA referenced in Table 1
[2] Name of phage mutated by CRISPY-BRED
[3] PAM site for each sgRNA
[4] sgRNA targeting sequence, followed by the strand targeted in parentheses; (+/-) indicates RNA transcripts are present on both strands

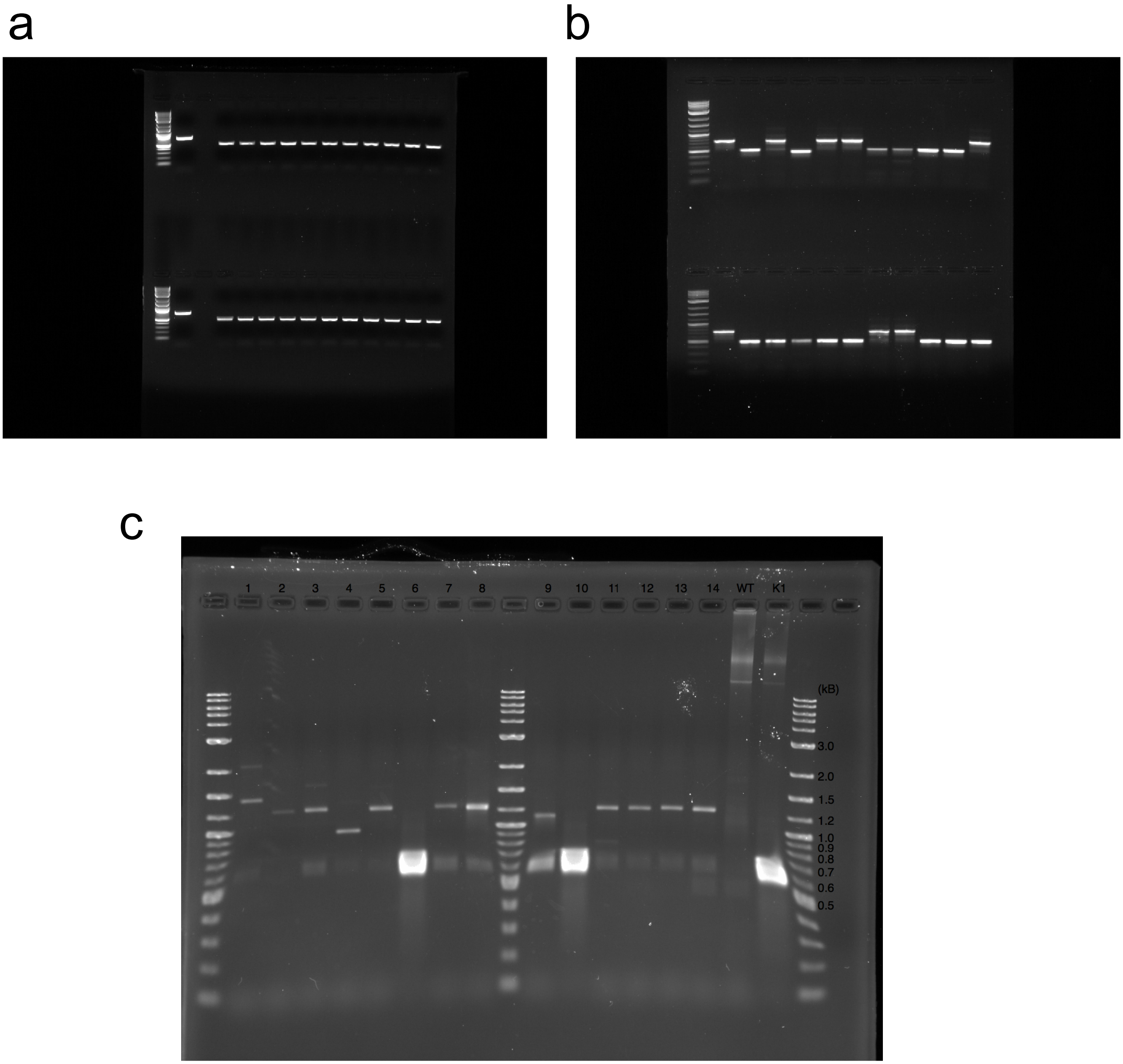

Supplementary Figure 1. Uncropped images of gels shown in Figures 1 and 2.
